# Prediction or interpretability?

**DOI:** 10.1186/s12982-019-0086-1

**Published:** 2019-07-10

**Authors:** Stefano Nembrini

**Affiliations:** Department of Pathology, Immunology and Laboratory Medicine, College of Medicine, Emerging Pathogens Institute, University of Florida, P.O. Box 100009, Gainesville, FL 32610-3633 USA

## Abstract

The journal published a review of the literature on recursive partition in epidemiological research comparing two decision tree methods: classification and regression trees (CARTs) and conditional inference trees (CITs). There are two sources of potential confusion in the paper for readers: one lies in the definition and the comparison of CITs and CARTs, while the other is more general and it refers to the use of hyper-parameters and their tuning through resampling techniques.

Venkatasubramaniam et al. [1] presented a very interesting paper on recursive partitioning in epidemiology. Their contribution is particularly relevant because they presented a novel graphical visualization tool that allows practitioners to identify subgroups.

Nevertheless, some of the statements therein contained are incorrect and might be misleading for non-experts in the field.

First of all, the paper conveys the idea that conditional inference trees (CITs) [2] are generally better than classification and regression trees (CARTs) [3] because they follow a *formal statistical inference procedure in each splitting step*, and only highlight the drawbacks of CART, while advocating for the use of CIT, because of their simplicity and ease of interpretation. While this definition can be found in the original paper by [2], readers who are not too familiar with the statistics and technicalities behind it, might draw the conclusion that CART is a less valid statistical method. CITs were born inside a *unbiased recursive partitioning framework*, with the aim of providing a unified *unbiased* splitting scheme by means of *p*-values in order to reduce *the effects of multiple comparisons in cutpoint selection* from which the original implementation of CART suffers [4, 5]. First noted that splitting criteria in CART favor predictors having more values (i.e. less missing values, more categories or distinct numerical values) and thus offering more split. This is particularly problematic in Random Forests, where trees are grown to purity, but is generally less relevant when single regression trees are grown, where stopping rules prevent uninformative splits [6]. Practitioners may feel more comfortable in using CITs because they are embedded in the familiar null *hypothesis significance testing* (NHST) framework. Nevertheless, we warn the readers that this ease comes at a cost: NHST has been criticized for decades (see for instance [7–10]) because it is not *even part of statistics proper*, and it is an *inconsistent hybrid of Fisher’s null hypothesis testing and Neyman*–*Pearson’s decision theory* [11].

The term *conditional* comes to the fact that the distribution of a response variable *Y* given the status of *m* covariates is being modeled by means of tree-structured recursive partitioning. It is assumed that the conditional distribution *D*(*Y |X*) of the response *Y* given the covariates *X* depends on a function *f* of the covariates *D*(*Y |X*) = *D*(*Y |f* (*X*_1_, …, *X*_*m*_)). In this sense, CART and generalized linear models [12] are also *conditional* models. Moreover, Venkatasubramaniam et al. [1] state that in CITs the association between each covariate and the outcome is quantified *using the coefficient in a regression model*.

This statement is wrong, because—according to [2]—dependence between *Y* and *X* is constructed by *means of the conditional distribution of linear statistics in the permutation test framework developed by* [13].

In the paper, it is also unclear where the conceptual differences between CART and CIT lie: CIT separates the selection of the split variable from selection of the split point of the already selected split variable, while CART does not. The association between *Y* and *X* is assessed through a linear rank test. For survival data, a log-rank transformation for censored data is performed. If the association is found to be significant, the covariate with minimal *p* value is selected for splitting and the optimal split point is found by comparing two-sample linear statistics for all possible partitions for the split variable. In addition to that, if a test statistic of quadratic form is used then splits are *unbiased* [14].

According to [15], two major disadvantages CITs are that the association test for selecting the split variable is based on linear rank statistics, while the optimal split is a dichotomous threshold-based split. Furthermore *linear* rank statistics cannot detect non-linear effects in the independent variables. An improvement on this procedure—while still providing unbiased split selection—was proposed by [15] and uses maximally selected rank statistics adjusted *p* values, so that split variable and split point selection happen at the same step, as in the original implementation of CART.

Another source of confusion in the paper lies in the **Stopping rules** section. Here, cross-validation (CV) is described as a *stopping rule* for recursive partitioning methods. Stopping rules are in fact based on hyper-parameters (usually referred to as *pre*-*pruning*) that control the complexity of the trees, both functions rpart and ctree in R share some, e.g. minbucket and minsplit, i.e. the minimum number of observations in any terminal node and the minimum number of observations that must exist in a node in order for a split to be attempted; while have others that are unique, i.e. rpart has a complexity parameter cp, e.g. the overall *R*^2^ must increase by cp at each step, while ctree has mincriterion, which is the 1 − *P* value that must be exceeded in order to implement a split, which is set to 0.95 by default. This is the purely conventional threshold used in NHST (i.e. *P* < 0.05), which might not be ideal for all applications [9]. The *sacred* 0.05 criterion was strongly discouraged by both Fisher [16] and Neyman–Pearson [17]. Setting cp in rpart to larger values (cp = 0.01 by default), might be enough to obtain smaller trees, avoiding interpretability problems and uninformative splits [6].

A common strategy is to grow a large tree with no more than a given number of samples in each node (e.g. minbucket = 5) [18], then this large tree is pruned (i.e. reduced) using *cost*-*complexity pruning*. All these parameters, refrain the tree from splitting further after the root, and can be evaluated through *cross*-*validation* (see for instance the caret package in R), which is sometimes referred to as *post*- *pruning*. The tree built with the set of hyper-parameters obtaining the smallest cross-validated error, or within a prespecified threshold, e.g. inside one standard error [3]. When CV is used for hyperparameter selection, a large number of trees over a grid of parameters have to be created and only one gets picked as a winner of that *competition*, while any tree can be pruned after it is generated. Since also mincriterion can be seen as a hyper parameter that is subject to optimization [2], it is not guaranteed that simpler trees—that can be obtained with a large mincriterion be necessarily the most appropriate for prediction, while also hyper-parameters in CART can be tuned in order to provide smaller and more interpretable trees. So the statement that CITs are preferable due to *simplicity and ease of interpretation* is due to a more stringent—although arbitrary—stopping rule set as default in the package (i.e. mincriterion = 0.95). This actually brings us back to a simple question: **Is prediction or interpretability what we are after?** Both CART and CIT can—and should be—cross-validated.

## Conclusions

This work had the aim of clearing up some incorrect and potentially confusing statements on recursive partitioning methods used in epidemiology contained in a paper published in the journal. Conditional inference trees usually provide simpler models compared to classification and regression trees just because the default settings in ctree are more stringent than those included in rpart. If the user requires rpart to provide simpler models, then more restrictive conditions on the splitting rules should be selected, since they will probably be less willing to interpret a tree with more than ten terminal nodes. When the focus is prediction, then hyper-parameters of both models should be optimized over a grid of values through some sort of re-sampling method, e.g. cross-validation or bootstrapping.

## Data Availability

Not applicable.

## Abbreviations

CART: classification and regression trees
CIT: conditional inference trees
CV: cross-validation
